# Biographies of Living Medical Men

**Published:** 1856-01

**Authors:** 


"*Biographies of Living Medical Men.*"—In our original department appears an article under the above heading, from the pen of an esteemed correspondent. Premising that we are assured beyond peradventure that this is not intended as a personal attack on any person, and that its author has no acquaintance with, or prejudice against the individual who is so unfortunate as to be chosen for an illustration, we shall proceed to offer a few thoughts in addition thereto.

In the individual case alluded to as immortalized in the New Jersey Medical Reporter, we recognize a gentleman who is undoubtedly deserving of the high praise he receives. But that does not imply the propriety of the publication of this "puff direct." Whether the subject of the laudation had any knowledge of the shower of celebrity about to descend upon him, we shall not discuss, but shall assume that the whole affair was an innocent instance of bad taste.

We should be inclined to suppose that the true explanation of the occurrence, is to be found in the fact that the gentleman in question had written an extremely valuable and interesting series of articles for the New Jersey Medical Reporter, that as a pendant to those articles he had also written a series of biographies of the Presidents of the American Medical Association (acting on the supposition that as they had attained to the highest office in the gift of the profession, they were fair subjects for comment, favorable or unfavorable) and that the editor of the "Reporter," feeling under great obligations to his correspondent, repaid him by this highly complimentary biography.

This is at any rate the fair and charitable view of the matter, and is probably the true one. Now we see only one reason why this should not be a correct way of complimenting a deserving man. If a man does a good thing, if by energy and genius he acquires a claim to immortality, it would be very pleasant and not at all injurious for him to receive the credit of it while living. The honors paid to eminent living men are a stimulus to good conduct in the rising generation. In this way good would result to community, and, moreover, if a man has earned fame, we do not see why he has not as good a right to the enjoyment of it as to the money which he has earned. Posthumous honors are but a sorry consolation for present neglect and penury.

But our "one reason" is a very serious objection to this pleasant theory. We will suppose that some one, stricken with admiration of Valentine Mott, writes a biography of the surgeon, which having general circulation adds to his business as well as tickles his vanity. Dr. Bodenhamer, the man who cures the piles, observing this, follows suite and procures a biography of himself, "beautifully illustrated" and profuse of compliments. This, also, is generally circulated, and enures to the benefit of Dr. Bodenhamer, giving him at once plenty of piles to treat, and a good large pile in his pocket, to the injury of better men.

Any man can get a biography written by applying to Mr. Something Livingston, of New York, who makes a business of it, and a profitable one too. In one of his volumes the whole family tree of our own tribe is duly immortalized. We never knew just what the job cost, but are assured that the thing is done cheaper now than then.

This, then, is our objection. As a general thing the temptation to lie gratuitously about a dead man is not great. We can in the main believe what is said about him, but as any living man may secure a biography to order (including a coat of arms at the Herald's Office) a suspicion is cast upon the veracity of this whole class of biographies, which very seriously impairs their market value, a fact which we have no doubt Mr. Livingston feelingly appreciates.

